# BRAF Modulates the Interplay Between Cell–Cell and Cell–Extracellular Matrix Adhesions in PECAM-1-Mediated Mechanotransduction

**DOI:** 10.3390/ijms252011234

**Published:** 2024-10-18

**Authors:** Éva Gráczer, Katalin Pászty, Laura Harsányi, Csilla Lehoczky, Antónia Fülöp, Andrea Varga

**Affiliations:** 1 Department of Biophysics and Radiation Biology, Semmelweis University, H-1094 Budapest, Hungary; graczer.eva@semmelweis.hu (É.G.); csokay.katalin@semmelweis.hu (K.P.);; 2 Faculty of Information Technology and Bionics, Pázmány Péter Catholic University, H-1083 Budapest, Hungary; 3 Faculty of Electrical Engineering and Informatics, Budapest University of Technology and Economics, H-1111 Budapest, Hungary

**Keywords:** actin cytoskeleton, BRAF RNAi, cell–cell adhesion, cell–ECM adhesion, endothelial monolayer, force application, magnetic beads

## Abstract

Mechanotransduction, the process of how cells sense and convert mechanical stimuli into biochemical response, is crucial in the migration of leukocytes or cancer cells through the endothelium during inflammation or metastasis. Migrating cells exert forces on the endothelium through cell surface adhesion molecules, such as platelet endothelial adhesion molecule PECAM-1, and this is essential for a successful transmigration. To study PECAM-1-mediated mechanotransduction, we applied PECAM-1-antibody-coated magnetic beads and exerted about 40 pN force on the endothelial monolayer. We show that force increases cell–ECM adhesion in the cell center and is accompanied by the opening of cell–cell junctions. Upon depletion of the MEK/ERK kinase, BRAF force increases cell–ECM adhesion both at the cell periphery and in the cell center, but this does not result in the opening of cell–cell junctions. Decreasing cell–ECM adhesion in BRAF-depleted cells through FAK inhibition results in the remodeling of cell–cell junctions. Force-induced increase in cell–ECM adhesion in the cell center correlates with the activation of the transcriptional cofactor Yes-associated protein (YAP). Furthermore, the induced activation of YAP through LATS inhibition prevents junctional remodeling in control cells. Thus, the activation of YAP might determine the strength of cell–cell junctions during PECAM-1-mediated mechanotransduction.

## 1. Introduction

Cell adhesion molecules are involved in cell–cell interactions and cell–extracellular matrix (ECM) interactions. Among them, platelet endothelial cell adhesion molecule 1 (PECAM-1) was identified as a mechanotransducer by forming a complex together with VE-cadherin and vascular endothelial growth factor receptor (VEGFR2), which, in response to flow, leads to the activation of Akt and eNOS [1,2,3,4]. As a result of this complex formation, actin stress fibers are formed parallel to the direction of the flow. Interestingly, in response to a reduced flow, the number of stress fibers is decreased, and actin gets reorganized to the cell periphery [5]. The importance of the same protein complex was recently identified in the transmigration of neutrophils. It was shown that neutrophils exert tension on PECAM-1 during their transendothelial migration (TEM) [6] and that leads to the ligand-independent activation of VEGFR2 to support TEM. Tension exerted through PECAM-1 was also investigated in a model system, where anti-PECAM-1 antibodies were attached to magnetic beads and tension was applied on bovine aortic endothelial cells [7]. It has been shown that force induced the tyrosine phosphorylation of PECAM-1 and the activation of ERK [7]. In a similar model system, with the activation of integrins and RhoA, focal adhesion growth and cytoskeletal stiffening was reported as a mechanoresponse [8]. However, these studies were conducted on sparsely cultured cells, and therefore the effect of tension could not be investigated on cell–cell adhesion. Tension exerted on other cell adhesion molecules, such as ICAM-1 [9], was also used to model ICAM-1 clustering (an event detected after binding of neutrophils to the endothelial cells [10]) and tensional forces exerted by neutrophils. The authors showed that tension resulted in an increased RhoA signaling and myosin light-chain phosphorylation in a LARG-dependent manner. VE-cadherin-based force transduction was shown to affect cell–ECM and cell–cell junctions [11,12,13]. Tension exerted on VE-cadherin resulted in the remodeling of cell–cell junctions and increased gap formation between endothelial cells [11]. In the same study, shear stress applied on PECAM-1-antibody-coated beads had similar consequences on cellular stiffening and intercellular gap formation. The authors suggested that focal adhesion kinase (FAK) couples integrin-mediated signals to force-activated cytoskeletal remodeling at stressed VE-cadherin complexes (at the bead–cell interface) [13] and this mechanism is ECM-dependent. Thus, tension exerted on different cell adhesion molecules can initiate similar signaling events, that will affect cell–extracellular matrix (ECM) signal transduction to strengthen cell–ECM adhesion, and this might influence (VE-cadherin-mediated) cell–cell adhesion.

Cell–cell and integrin-mediated cell–ECM adhesions are linked through the actin cytoskeleton and share signaling molecules. Their coordinated interplay can be important for maintaining tensional homeostasis [14], and it might also be important for maintaining/restoring the barrier function of the endothelium during leukocyte or cancer cell TEM. A possible model proposed for endothelial barrier stabilization is that cell–cell and cell–ECM adhesions at the cell periphery work together to counteract the internal tension caused by actomyosin contraction [15]. Indeed, leukocytes exert tension on the endothelial monolayer and induce actomyosin contraction during TEM [16]. Thus, the local interplay between cell–cell and cell–matrix adhesion might determine the efficiency of TEM and it might be possible that the local regulation rather than a global manipulation of cell–matrix adhesion could be a potential strategy to influence the barrier function of the endothelium or a successful TEM.

ECM stiffness can be sensed by endothelial cells through integrins [15]. Integrin activation through a protein complex composed of FAK, vinculin, and talin (as well as other actin-binding proteins and actin itself) can result in the reorganization of the actin cytoskeleton. Activation of FAK can activate the Hippo pathway component, Yes-associated kinase (YAP) or inactivate its upstream kinase, LATS1/2 [17]. Activation of YAP (elevating its nuclear localization) is also regulated by the remodeling of the actin cytoskeleton and correlates with an increased cell stiffness [18]. The question arises whether force-induced integrin activation and actin reorganization affect YAP activity and whether this plays a role in the regulation of cell–cell adhesion. It has been shown that YAP nuclear localization is enhanced upon thrombin treatment, and this might have a role in barrier repair after thrombin treatment [19]. YAP also promotes endothelial barrier repair after TNFα-induced injury [20]. Conversely, monolayer permeability is increased in siYAP/siTAZ knockdown endothelial cells [21].

We have previously shown that the MEK/ERK kinase, BRAF supports thrombin-induced permeability of human umbilical vein endothelial cells (HUVECs) and in vitro transmigration of A375 melanoma cells through the HUVEC monolayer [22]. This effect of BRAF on the VEGF- or thrombin-induced permeability is not related to its function as a MEK/ERK kinase, since permeability defects cannot be detected by MEK inhibition [23,24]. Rather, BRAF, through its heterodimerization with RAF1, can affect the reorganization of the actin cytoskeleton and regulates paracellular permeability. In vivo, endothelial-restricted BRAF knockout mice [23] show a reduced extravasation of B16F10 melanoma cells in the lung vasculature after tail vein injection, and that might indicate the role of BRAF in the regulation of metastatic spreading. Since BRAF modulates stress fiber formation and the reorganization of the actin cytoskeleton in response to thrombin [22] and this influences cell stiffness, the question arises whether BRAF, through the modulation of the actin cytoskeleton dynamics, might be part of a mechanotransduction pathway specifically activated through a cell adhesion molecule. To answer this question, here we used a model system where about 40 pN force was applied on endothelial PECAM-1 by using PECAM-1-antibody-coupled magnetic beads and a permanent magnet [25]. By using this model system, we analyzed cell–cell and cell–ECM adhesion upon force application, and we studied the role of BRAF therein by siRNA knockdown. We found a correlation between cell–ECM adhesion and YAP activity in the context of PECAM-mediated mechanotransduction. BRAF depletion resulted in an increased activity of YAP, and the barrier function of the cells was strengthened. Force application reduced the barrier function of control cells, which could be restored by YAP activation.

## 2. Results

### 2.1. Force-Induced Stress Fiber Formation Is Reduced in BRAF-Depleted Cells

**Figure 1:**
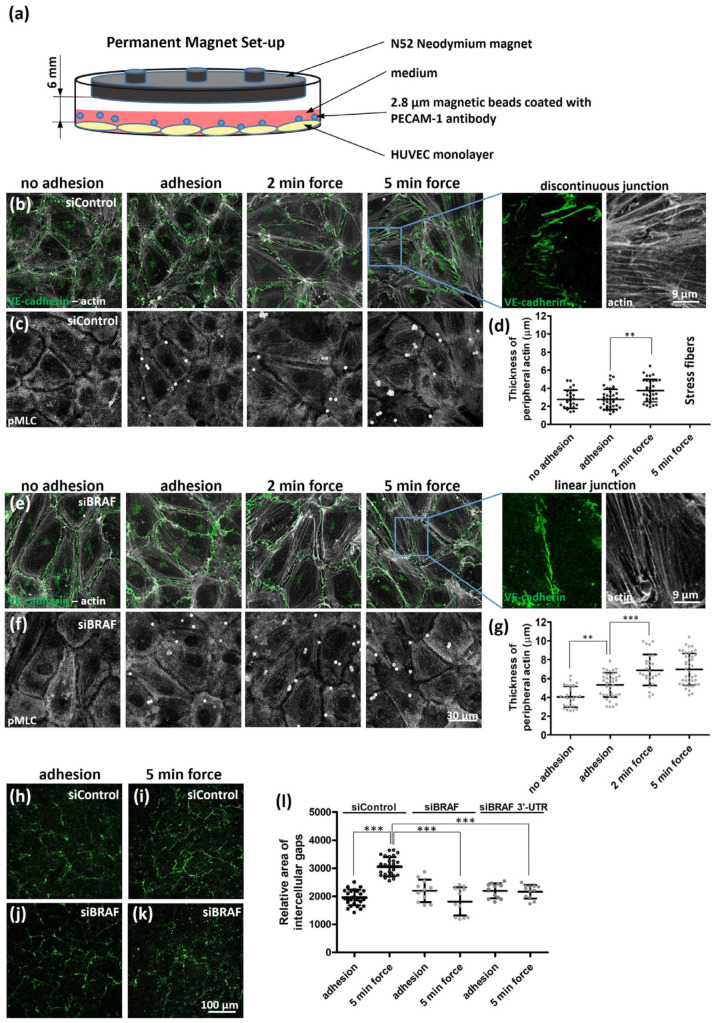
Force-induced stress fiber formation is reduced in BRAF-depleted cells. (**a**) The experimental setup of neodymium magnets to induce mechanotransduction through the cell surface receptors by magnetic force. The cells were grown on a 3.5 cm culture dish and the magnet was fixed to the lid at a distance of about 6 mm from the cells, which makes the field strength constant (~40 pN) and permits control of the time of exposure to the magnetic field. Immunofluorescence images of siControl- (**b**,**c**) and siBRAF-transfected monolayers (**e**,**f**) are shown without adhesion of anti-PECAM-1 beads or with adhesion either before or after application of force for two and five minutes. VE-cadherin (green) and actin (grey) staining are merged to analyze the thickness of peripheral actin on panels (**b**,**e**). The location of pMLC is illustrated in panels (**c**,**f**). White circular structures on panels (**c**,**f**) show the magnetic beads, where PECAM-1 antibody on the bead surface is stained with the same secondary antibody as pMLC. Enlarged images of VE-cadherin and actin show an example of the remodeling (discontinuous) junction for siControl cells after five-minute force application, and the linear (continuous) junction for siBRAF cells after application of five-minute force. Quantification of the “apparent” thickness of peripheral actin in siControl (**d**) and siBRAF (**g**) samples is presented without adhesion or with adhesion either before or after application of two- and five-minute force. Results are shown from two independent experiments of cells with no treatment and from three independent experiments for all dataset with adhesion or force application and were carried out at least with two different LOTs of HUVECs. Immunofluorescence images of intercellular gaps of (**h**,**i**) siControl- and (**j**,**k**) siBRAF-transfected cells with adhesion (**h**,**j**) or upon force application (**i**,**k**). (**l**) Quantification of the relative area of intercellular gaps is shown in (**h**–**k**). The results are from three independent experiments of control cells (both for adhesion and force application) and one experiment with siBRAF and another experiment with siBRAF 3′-UTR. Data were analyzed by one-way ANOVA (**d**,**g**) or three-way ANOVA (**l**) followed by Bonferroni’s multiple comparison test expressed as mean ± SD (** *p* < 0.01, and *** *p* < 0.001).

To understand how mechanical force exerted through PECAM-1 regulates the cellular response of HUVECs, we used PECAM-1-antibody-conjugated magnetic beads, and by using a permanent magnet, we exerted about 40 pN force [25] on the HUVECs for different time intervals (see Figure 1a for the experimental set-up). We wanted to investigate how a confluent monolayer—as a model of endothelium—reacts to the applied external force; therefore, we followed the localization changes in actin (together with the cell–cell adhesion molecule, VE-cadherin, Figure 1b) and the phosphorylated myosin light chain (pMLC, Figure 1c). We studied not only the effect of force but also adhesion itself. We did not observe any significant thickening of the peripheral actin ring upon PECAM-1-mediated adhesion. However, we could confirm the increased “apparent” thickness of the actin ring (determined by the line plot analysis of the fluorescence intensity of actin from confocal images, as shown in Supplementary Figure S1a–d, blue arrows) upon force application (quantification is shown in Figure 1d, Supplementary Figure S1a–d) as published for single cells [8]. More importantly, when we exerted force for 5 min through PECAM-1, stress fibers were formed and VE-cadherin was reorganized at several cell–cell junctions from linear to focal adherens junctions, featuring an interrupted (discontinuous) VE-cadherin pattern. Localization of pMLC followed the pattern of actin staining (Figure 1c).

To understand whether BRAF has a role in the force-induced reorganization of the actin cytoskeleton through PECAM-1, we depleted BRAF by siRNA and compared the localization of actin (and VE-cadherin Figure 1e) and pMLC (Figure 1f) to the changes observed in control cells. As it has been shown, BRAF-deficient cells form a thicker peripheral actin ring than control cells (Figure 1d,g and Supplementary Figure S1a–h). Interestingly, the “apparent” thickness (Supplementary Figure S1e–h, blue arrows) of the actin ring increased in BRAF-depleted cells already upon adhesion and increased further upon force application. We did not observe discontinuous VE-cadherin junctions in BRAF-depleted cells upon force application (see enlarged images of siControl and siBRAF cells after five-minute force application in Figure 1). pMLC staining followed the pattern of actin staining (Figure 1f).

We could verify these data by using a different set of siRNAs targeting the 3′-UTR region of BRAF mRNA sequence where there was also no reorganization of the peripheral actin ring into stress fibers upon force application in BRAF-depleted cells. (Supplementary Figure S1i–l). The efficiency of the two different siRNA sets was confirmed by Western blotting (Supplementary Figure S1m). In order to rescue the phenotype, we used the 3′-UTR siRNA to silence endogenous BRAF and transduced the cells to re-express EGFP-BRAF. Although the transduction efficiency was low, the cells expressing EGFP-BRAF showed remodeling of (discontinuous) VE-cadherin junctions (Supplementary Figure S1n) and stress fiber formation upon force application (Supplementary Figure S1o,p).

To examine whether VE-cadherin reorganization is followed by intercellular gap formation upon force application, we plated the cells on biotinylated gelatin and after the adhesion, or force application, fixed the cells and stained them with Alexa488-conjugated avidin to label intercellular gaps connecting the discontinuous intercellular junctions. We observed a significant increase in the Alexa488-labeled area of control (Figure 1h,i, quantification in Figure 1l) but not that of BRAF-depleted cells (Figure 1j,k, quantification in Figure 1l).

To investigate whether the different patterns of pMLC staining are reflected in the phosphorylation of MLC, we compared pMLC levels in control and BRAF-depleted cells upon adhesion and force application (Supplementary Figure S1q,r). Adhesion did not induce MLC phosphorylation in either case. Five-minute force application led to a significant increase in MLC phosphorylation in control cells, in accordance with what was found in bovine aortic endothelial cells [8]. BRAF depletion reduced the extent of force-induced MLC phosphorylation.

Taken together, force application through PECAM-1 resulted in the reorganization of VE-cadherin junctions in the control but not in the BRAF-depleted HUVECs. In line with this observation, VE-cadherin reorganization is followed by intercellular gap formation only in control cells. MLC phosphorylation upon force application is reduced upon BRAF knockdown.

### 2.2. Inhibition of BRAF Dimerization or MEK Activity Cannot Phenocopy the Effect of BRAF Depletion

**Figure 2:**
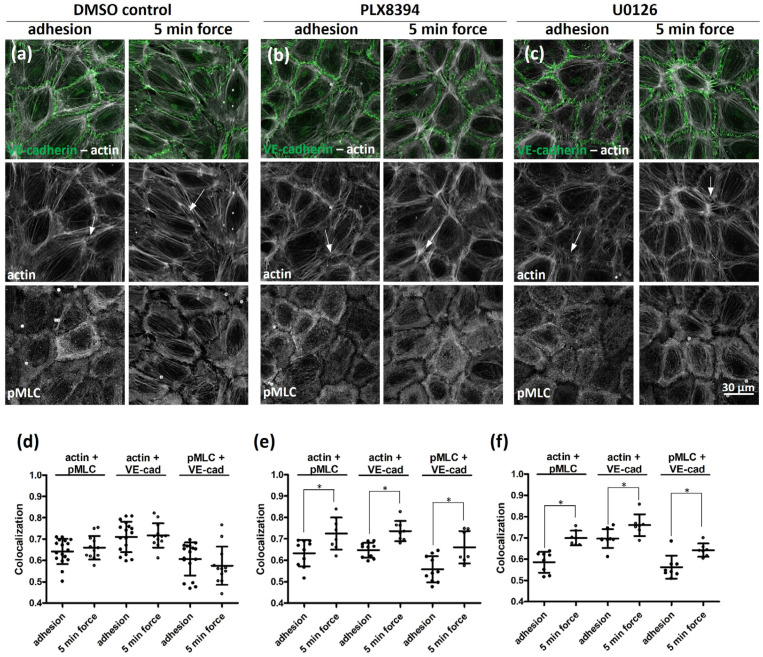
Inhibition of BRAF dimerization or MEK activity cannot phenocopy the effect of BRAF depletion. Localization of actin (grey), VE-cadherin (green), and pMLC (grey) of HUVEC monolayers treated with vehicle (DMSO) (**a**); BRAF-specific inhibitor PLX8394 (1 µM, 1 h) (**b**) and MEK-specific inhibitor U0126 (10 µM, 1 h) (**c**) were analyzed either before or after five minutes of force application. White arrows indicate the remodeling (discontinuous) junctions. Enlarged images of these junctions are presented in Supplementary Figure S2k–p. For quantification of colocalization of actin and pMLC or actin and VE-cadherin (VE-cad) or pMLC and VE-cadherin (VE-cad) in (**d**) DMSO-, (**e**) PLX8394-, or (**f**) U0126-treated cells, the Colocalization Colormap plugin in ImageJ was used. This plugin calculates an index of colocalization where 1 stands for complete colocalization and 0 means lack of colocalization. Results are shown from two independent experiments and were carried out with two different LOTs of HUVECs. Data were analyzed by two-way ANOVA followed by Bonferroni’s multiple comparison test, expressed as mean ± SD (* *p* < 0.05).

To understand whether the effect of BRAF depletion is dependent on its heterodimerization with RAF-1 or is related to its activity on MEK, we compared the localization of actin (and VE-cadherin) and of pMLC in DMSO-treated (control) cells (Figure 2a), as well as in the presence of the dimerization inhibitor PLX8394 (Figure 2b) or the MEK inhibitor U0126 (Figure 2c). We have chosen the concentration of PLX8394 not to affect basal ERK phosphorylation (Supplementary Figure S2q,s), only heterodimer formation between BRAF and RAF1. At this concentration, PLX8394 reduced heterodimer formation between BRAF and RAF1 by 50% determined by immunoprecipitation (Supplementary Figure S2a). U0126 treatment efficiently abrogated the activity of MEK, which was confirmed by comparing ERK phosphorylation of DMSO and U0126-treated cells (Supplementary Figure S2b). In DMSO-treated cells force application resulted in the appearance of actin stress fibers, and discontinuous VE-cadherin junctions (Figure 2a and Supplementary Figure S2c–f). In PLX8394 or U0126 inhibitor-treated cells, force induced the appearance of so-called VE-cadherin invaginations (Figure 2b,c, Supplementary Figure S2c–j) (reviewed in [26]), which were also observed upon VEGF and ROCK inhibitor treatments by others [27]. In addition, we detected discontinuous VE-cadherin junctions, where two neighboring cells were connected through radial actin fibers (Figure 2a–c and Supplementary Figure S2k–p), which were attached to peripheral actin rings of the two neighboring cells. These kinds of structures were previously identified in epithelial cells, when they formed cell–cell contacts in sparse cultures [28]. To further analyze the composition of these remodeling junctions, we performed a line plot analysis to investigate the actomyosin activity at these junctions. Interestingly, in DMSO-treated cells (Supplementary Figure S2k,l), actin colocalizes either with pMLC or with VE-cadherin but not with both. However, in the case of either inhibitor treatment all three were found rather together at the junctions (Supplementary Figure S2m–p). The presence of pMLC at these VE-cadherin junctions upon inhibitor treatments indicates that these junctions are under tension. Indeed, force application in the presence of the inhibitors significantly increased the colocalization of pMLC with actin (or with VE-cadherin) (Figure 2d–f) at cell–cell junctions.

To find out whether these local changes also appear globally, we checked the phosphorylation of MLC upon adhesion and force application both in the absence and in the presence of PLX8394 and U0126 inhibitors (Supplementary Figure S2q). The phosphorylation of MLC was increased upon force application in DMSO-treated cells (Supplementary Figure S2q,r), but not in the presence of PLX8394. U0126 treatment slightly decreased the level of pMLC upon force application. We also determined pERK levels, since this was shown to increase in PECAM-1-mediated force application [7,8], and checked the effect of the inhibitors therein. The quantitative analysis showed (Supplementary Figure S2s) that in DMSO-treated cells, pERK level was increased to 1.5-fold upon force application. PLX8394 treatment did not significantly affect the basal pERK level upon adhesion but decreased the force-induced response. U0126 inhibited ERK phosphorylation and did not significantly increase the force-induced response.

To correlate the observed changes in the inhibitor-treated monolayers with the efficiency of A375 melanoma migration through the inhibitor-pretreated HUVEC monolayers, we measured the transmigration efficiency in the absence and in the presence of thrombin and the specific inhibitors (Supplementary Figure S2t). Surprisingly, HUVEC monolayers treated with either PLX8394 or U0126 showed an increased number of migrated A375 cells compared to the DMSO-treated cells. In the presence of thrombin, all conditions showed similar numbers of transmigrated A375 cells.

These data indicate that a special actin organization can be found in those remodeling junctions, which were observed in epithelial sheets upon adhesion belt formation but rarely can be seen in remodeling junctions of endothelial cells. The observed remodeling junctions in inhibitor-treated HUVEC cells are consistent with an increased number of melanoma cells migrated through these HUVEC monolayers.

### 2.3. BRAF Depletion Increases the Number and Distribution of pFAK Positive Cell–ECM Adhesion Sites

**Figure 3:**
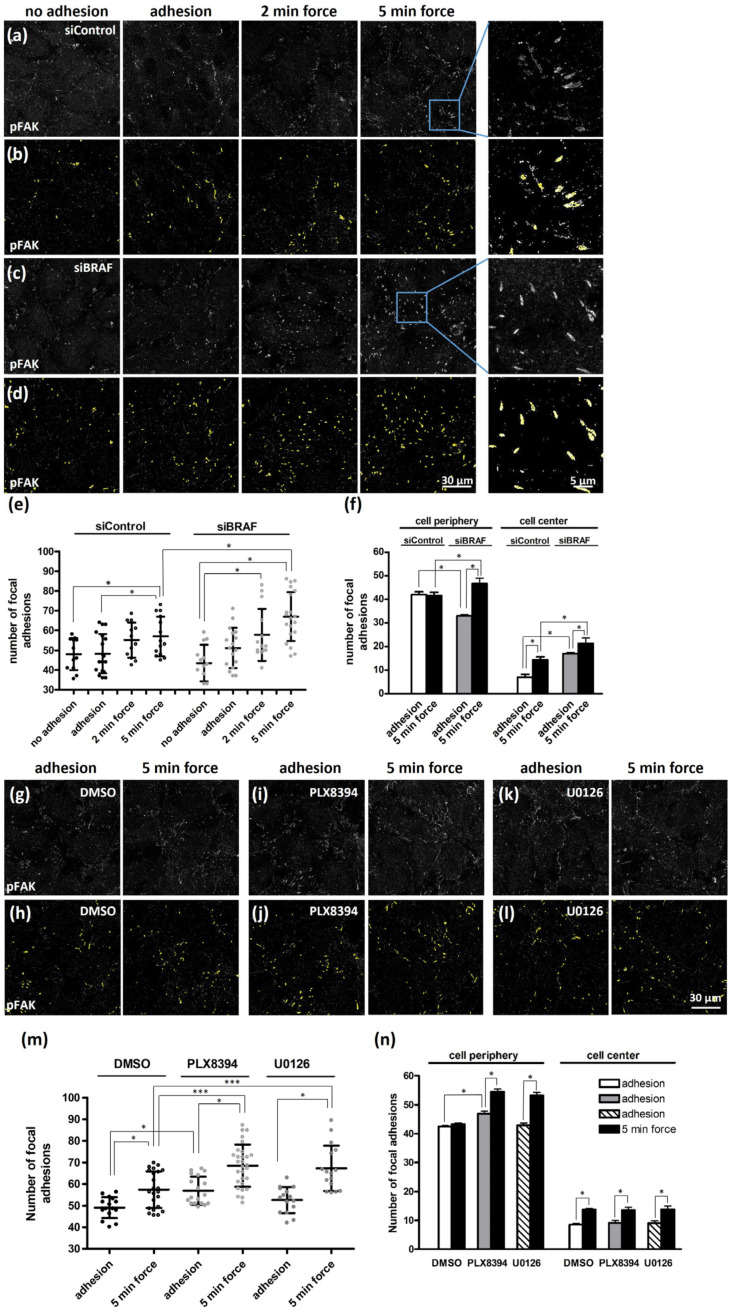
BRAF depletion increases the number and distribution of pFAK-positive cell–ECM adhesion sites. siControl- (**a**,**b**) and siBRAF-transfected (**c**,**d**) HUVEC monolayers left untreated (no adhesion) or exposed to either adhesion alone or to two- and five-minute force applications were fixed and stained for pFAK. The number of pFAK spots in each field of view (120 × 120 μm area) was determined as illustrated in yellow for siControl- (**b**) and siBRAF-transfected (**d**) cells. Enlarged images at the end of each row show the focal adhesions determined after five-minute force application. The sum of the quantification (determined for each field of view) is demonstrated on panel (**e**). The number of pFAK spots was also examined in 5 µm vicinity of the cell periphery and at the cell center and are presented in panel (**f**). The numbers show the sum of pFAK spots determined in each field of view, separately showing the sum of pFAK spots at the cell periphery and center. The number of pFAK spots on HUVEC monolayers treated with vehicle (DMSO) (**g**,**h**); BRAF-dimerization inhibitor PLX8394 (1 µM, 1 h) (**i**,**j**) or MEK inhibitor U0126 (10 µM, 1 h) (**k**,**l**) were determined either before or after five minutes of force application. Panel (**m**) displays the quantification of pFAK spots in the presence or absence of inhibitors. The number of pFAK spots is also determined in 5 µm vicinity of the cell periphery and at the cell center in the presence of PLX8394 or U0126 (**n**). Representative images from three independent experiments are shown. Data were analyzed by two-way ANOVA, followed by Bonferroni’s multiple comparison test, expressed as mean ± SD (* *p* < 0.05, and *** *p* < 0.001).

To elucidate the connection between the strength of cell–cell and cell–ECM junctions, we analyzed FAK activation upon PECAM-1-mediated adhesion and force application by quantifying the fluorescence signal of autophosphorylated FAK. In accordance with previous data [8], force application through PECAM-1 significantly increased the number of focal adhesions (Figure 3a,b,e).

We also investigated how BRAF depletion (Figure 3c–e) influences cell–ECM adhesion through FAK autophosphorylation. We found that BRAF depletion increased the number of focal adhesions already after two minutes of force application, which was not significant in control cells. When we analyzed the distribution of focal adhesions (Supplementary Figure S3), we found that in control cells there were less focal adhesions in the cell center and more at the cell periphery compared to BRAF-depleted cells (Figure 3f). Upon force application, the number of focal adhesions increased in the cell center of control cells, but their number was still less than what we found in BRAF-depleted cells. At the cell periphery of control cells, no increase in the number of focal adhesions was detected in the presence of force, while BRAF-depletion significantly increased it.

Our next question was how the number or localization of pFAK might be regulated by the inhibition of BRAF dimerization or MEK activity. Therefore, we compared the DMSO-treated (Figure 3g,h), PLX8394- (Figure 3i,j) and U0126-treated (Figure 3k,l) HUVEC cells. The analysis showed an increased number of focal adhesions in all cases under force, but the increase was more pronounced upon the inhibitor treatments and the extent was similar to the numbers observed upon BRAF depletion (Figure 3m). To find out how the distribution of pFAK-positive cell–ECM adhesion sites changed upon inhibitor treatment, we analyzed the changes observed upon force application at the cell periphery or in the cell center (Figure 3n) and found that in the cell center the numbers were very similar in all cases, and thus the increased total number of adhesion sites stemmed from the increase at the cell periphery. 

Altogether, the application of force increases the number of pFAK-positive cell–ECM adhesion sites in all conditions. However, BRAF-depleted cells differ from control cells and inhibitor-treated cells in such a way that BRAF-depleted cells have more focal adhesions in the cell center already upon adhesion and this number increases further upon force application, giving the highest absolute number of focal adhesions in the cell center. The number of focal adhesions is increased at the cell periphery in all cases, but not in control cells.

### 2.4. FAK Inhibition Results in the Weakening of Cell–Cell Junctions in BRAF-Depleted Cells

**Figure 4:**
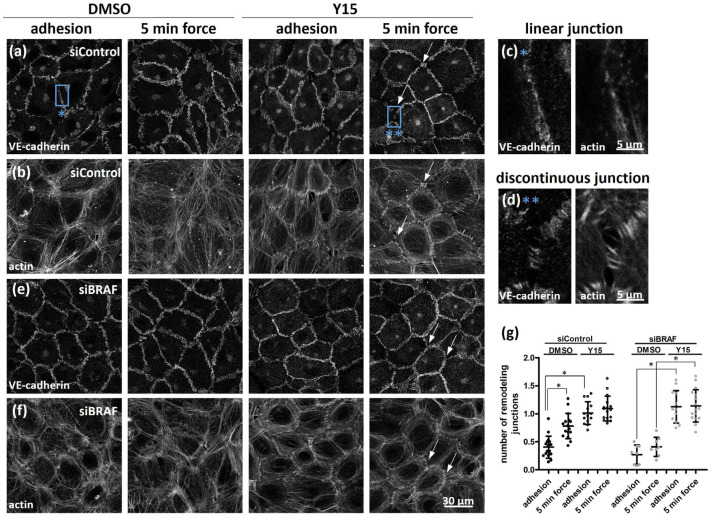
FAK inhibition results in the weakening of cell–cell junctions in BRAF-depleted cells. The effect of the pFAK-specific inhibitor, Y15 (15 μM, 1 h) was analyzed on the phenotype of VE-cadherin (**a**,**e**), or on the localization of actin (**b**,**f**) in control (**a**,**b**) and BRAF-depleted (**e**,**f**) cells either when the anti-PECAM-1-coated beads adhered to the cells or after further force application. White arrows indicate discontinuous VE-cadherin-labeled cell–cell contacts. Representative images from two independent experiments are shown. Blue boxes on siControl images are enlarged and illustrate the linear VE-cadherin junctions and the actin staining in siControl cells with adhesion (**c**, blue asterisk) and discontinuous VE-cadherin junctions and the actin staining in Y15-treated siControl cells after force application (**d**, two blue asterisks). Panel (**g**) shows the relative number of remodeling (discontinuous) junctions. Data were analyzed by two-way ANOVA, followed by Bonferroni’s multiple comparison test expressed as mean ± SD (* *p* < 0.05).

To explore whether strengthening of cell–ECM adhesion upon force application influences cell–cell adhesion, we used a specific inhibitor that prevents FAK autophosphorylation on residue Tyr397 (Y15). The effect of FAK inhibitor treatment reduced the phosphorylation of FAK by about 60%, as confirmed by Western blotting (Supplementary Figure S4). We investigated how this inhibitor affected the organization of VE-cadherin (Figure 4a) and actin (Figure 4b) in control cells. Interestingly, FAK inhibition resulted in a similar “adhesion belt” formation that we found upon PLX8394 and U0126 treatments, and this was accompanied with discontinuous junction formation (Figure 4c—linear junction and Figure 4d—discontinuous junction). To find out whether the increased number of force-induced pFAK-positive adhesions in BRAF-depleted cells has a role in the stabilization of cell–cell junctions, we treated BRAF-depleted cells with the Y15 inhibitor. Indeed, we could observe similar patterns of VE-cadherin (Figure 4e) and actin (Figure 4f) localization like those in control cells treated with the FAK inhibitor. Thus, weakening of cell–ECM adhesion by FAK inhibitor treatment results in the remodeling of cell–cell junctions in both control and BRAF-depleted cells (see the quantified relative number of remodeling junctions in Figure 4g).

### 2.5. YAP Activation in Control Cells Prevents the Force-Induced Remodeling of VE-Cadherin Junctions

**Figure 5:**
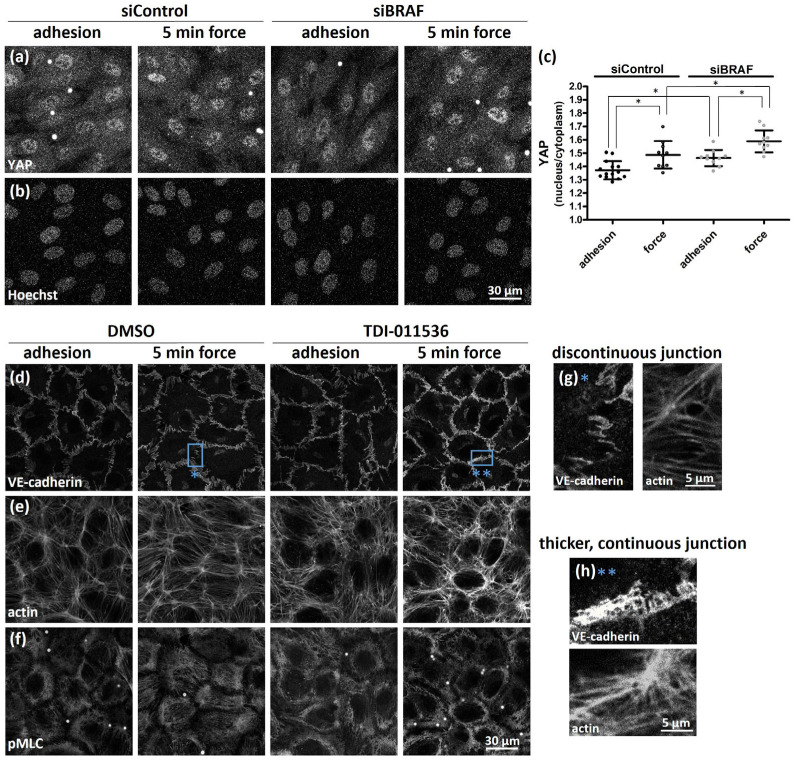
The extent of YAP nuclear localization correlates with increased cell–ECM adhesion in the cell center. YAP localization was investigated on siControl- and siBRAF-transfected HUVEC monolayers with either anti-PECAM-1-coated magnetic beads to mimic adhesion or with further five-minute force application (**a**,**b**). The quantification of YAP distribution between nucleus and cytoplasm is demonstrated in panel (**c**). Data were analyzed by two-way ANOVA (**c**) followed by Bonferroni’s multiple comparison test expressed as mean ± SD (* *p* < 0.05). To increase the activity of endogenous YAP, a LATS inhibitor, TDI-011536 (5 μM, 1 h), was used, and its effect was analyzed on the localization of VE-cadherin (**d**), actin (**e**), and pMLC (**f**). Representative images from two independent experiments are shown. Blue boxes illustrate either the discontinuous VE-cadherin junctions and the actin staining in DMSO-treated cells after force application (**g**, blue asterisk) or thicker, continuous VE-cadherin junctions and the actin staining in LATS inhibitor-treated cells after force application (**h**, two blue asterisks).

To correlate the observed changes in cell–ECM adhesion with YAP activation, we investigated whether force application through PECAM-1 increases YAP activation. In control cells, as expected, force application increased YAP nuclear translocation (Figure 5a–c). Interestingly, YAP activity was already increased upon adhesion in BRAF-depleted cells (Figure 5a–c) and increased further upon force application. While the numbers of total pFAK-positive cell–ECM adhesions in control and BRAF-depleted cells were similar upon PECAM-1 mediated adhesion (Figure 3e), the extent of YAP nuclear localization was different (Figure 5c). However, we found a positive correlation between YAP nuclear localization (Figure 5c) and the number of cell–ECM adhesions in the cell center of control and BRAF-depleted cells (Figure 3f).

To investigate whether YAP activity can influence cell–cell junctional remodeling upon force application, we treated control cells with the LATS inhibitor TDI-011536 to increase YAP activity. The effect of the inhibitor on YAP Ser127 (inhibitory) phosphorylation is shown in Supplementary Figure S5. While in control cells treated with DMSO, discontinuous VE-cadherin junctions and the actin staining (Figure 5d,g) are visible (and a similar pMLC pattern, Figure 5f) after force application, this was not seen in the inhibitor-treated cells (Figure 5d–f). Rather, peripheral actin bundles run parallel to the VE-cadherin staining, a similar pattern observed in BRAF-depleted cells without inhibitor treatment. The VE-cadherin staining, however, was different from the thinner, linear structures of BRAF-depleted cells (and also from the discontinuous junctions observed with U0126 or PLX8394 treatment). LATS inhibitor treatment induced a thicker but not discontinuous VE-cadherin junction pattern (Figure 5h).

These data indicate that YAP activity is increased upon PECAM-1-mediated mechanotransduction and the activation correlates with the increased number of cell–ECM adhesion sites in the cell center but not at the cell periphery. Furthermore, by increasing YAP activation in control cells, force application failed to induce the formation of discontinuous VE-cadherin cell–cell junctions.

## 3. Discussion

Transmigration of leukocytes and cancer cells through the endothelial monolayer can be viewed as a process regulated not only by biochemical but also biomechanical signaling. It has been shown that both leukocytes and vascular endothelial cells exert three-dimensional forces to facilitate the transmigration of leukocytes [16]. Quantification of the forces measured by using leukocytes and ICAM-1-coated beads pointed out that similar traction forces are exerted by both the leukocyte and the bead. They consisted of a central rounded region of downward pushing surrounded by a weaker rim of upward pulling underneath the leukocyte or the bead. However, how mechanical signals are translated into biochemical signaling that regulates the interplay between cell–cell and cell–ECM adhesion is less investigated. Here we show by using a model system that mechanical signal through the adhesion molecule PECAM-1 results in tension-mediated disruption of cell–cell adhesion. This process is accompanied by an increase in cell–ECM adhesion in the cell center of the endothelial cells and it correlates with the activation of the transcriptional regulator YAP. Indeed, tension exerted by a neutrophil through PECAM-1 is required for its successful TEM [6] and this might be relevant in cancer cell TEM, too.

We have shown previously that depletion of the MEK/ERK kinase, BRAF decreases the permeability of the endothelial monolayer and the transmigration of cancer cells in response to thrombin and this is related to the role of BRAF exerted on the dynamical reorganization of the actin cytoskeleton [22]. Since tension sensing by PECAM-1 is required for a successful TEM [6], we wanted to use our model system to analyze whether BRAF depletion has a role in PECAM-1-mediated mechanotransduction. We have found that BRAF depletion affects the distribution of cell–ECM adhesion sites: more cell–ECM adhesion sites are localized in the cell center and fewer are found at the cell periphery in the absence of force. Force application significantly increases the number of cell–ECM adhesion sites in the cell center of control cells but not at their periphery. In BRAF-depleted cells the force-induced increase is localized to both the cell periphery and the cell center. We wanted to gain mechanistic insight into how BRAF depletion exerts this effect on melanoma TEM and actin reorganization. Therefore, we used PLX8394 to inhibit BRAF homodimerization and heterodimer formation with RAF1 [29]. We show here that the 50% decrease in BRAF-RAF1 heterodimer formation does not phenocopy BRAF depletion in terms of melanoma TEM. However, we could show that although the extent of the overall increase in the number of cell–ECM adhesion sites is similar in BRAF-depleted cells and PLX8394-treated cells, the distribution of cell–ECM adhesion sites is different. Upon adhesion, the number of cell–ECM adhesion sites in the cell center was similar to control cells, but lower than in BRAF-depleted cells. The cell–ECM adhesion was strengthened at the cell periphery in the inhibitor-treated cells upon force application, and a similar trend was obtained in BRAF-depleted cells. In line with the results of our melanoma TEM experiments we could see remodeling junctions in the inhibitor-treated cells, although these were found to be under continuous tension. These junctions were different from the remodeling junctions observed in DMSO-treated cells; they resembled the adhesion belt of epithelial cells [28], which might be dependent on the CAMKK2/AMPK pathway [30]. It was reported that the activity of ERK, downstream of MEK, might regulate the localization and the activity of AMPK [31]. Whatever mechanism operates in the presence of the inhibitor, it is conceivable that although pMLC levels are not increased upon force application in inhibitor-treated cells, pMLC is localized to VE-cadherin-actin structures and myosin-mediated tension might help to close these junctions. It is possible that this might be due to an increased ROCK activity at VE-cadherin junctions [23] upon decreasing BRAF dimer formation. In the presence of the MEK inhibitor U0126, we could see a very similar pattern of pMLC in the remodeling VE-cadherin junctions. The overall levels of pMLC did not increase here upon force application, which might be due to the lack of ERK activity (downregulated by U0126 treatment) on myosin light-chain kinase [32]. Thus, by using the inhibitors, we could conclude that they cannot phenocopy the effect of BRAF depletion. It is possible that upon PLX8394-inhibitor treatment the presence of 50% heterodimers is still enough to remodel VE-cadherin junctions, but with a phenotype which is different from what we found in control cells (i.e., no stress fibers are visible). It is also possible that PLX8394 (similar to vemurafenib) might have off-target effects, which could account for the disruption of cell–cell junctions [33,34]. The question arises what mechanism could serve for the different distribution pattern of pFAK upon BRAF depletion. One possible explanation would be that the amount and/or the localization of Rho-GTP is changed upon BRAF depletion, which we have previously found upon VEGF treatment [23]. The difference between the BRAF-depleted and the inhibitor-treated cells is the number of cell–ECM adhesion sites in the cell center. Importantly, these numbers correlate with force-induced YAP activation. Thus, YAP activation status might be an important indicator to predict how PECAM-1-mediated force affects cell–cell junctions. Moreover, by inducing YAP activation in control cells (through LATS inhibition), the force-induced remodeling of VE-cadherin junctions is prevented; thus, the strength of cell–cell adhesion can be modulated through the activity of YAP in a Hippo-pathway-dependent manner.

It has been shown that FAK inhibition affects cell–cell junctions differently (weakens or strengthens) depending on the experimental contexts (conditions, cell types) [15]. Since force application through PECAM-1 activates FAK and results in discontinuous junctions, we wanted to address how inhibition of focal adhesion (i.e., weakening of cell–ECM adhesion) affects cell–cell junctions. Decreasing cell–ECM adhesion still resulted in discontinuous junctions in control cells. More importantly, FAK inhibition in BRAF-depleted cells reorganized cell–cell junctions. Thus, a decrease in cell–ECM adhesion weakens the cell–cell adhesions and this crosstalk is modulated through BRAF.

**Figure 6:**
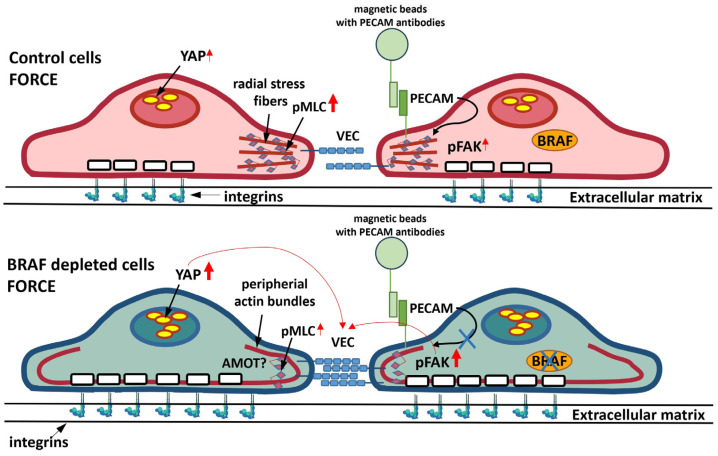
Schematic representation of the effect of BRAF depletion on FAK phosphorylation and YAP activity. In control cells, force acting through PECAM-1 induces radial actin stress fiber formation and this results in discontinuous VE-cadherin junctions. Cell–ECM adhesion is increased in the cell center and this correlates with an increased nuclear localization of YAP. BRAF depletion increases the thickness of peripheral actin bundles and does not perturb VE-cadherin junctions. Cell–ECM adhesion is strengthened further in the absence of BRAF compared to control cells and this leads to an increased translocation of YAP to the nucleus, which might strengthen cell–cell junctions.

Taken together, we could find a correlation between the stability of cell–cell junctions and an increased cell–ECM adhesion obtained in the cell center but not at the cell periphery. Increased cell–ECM adhesion in the cell center correlates with YAP activation (Figure 6) and increased YAP activation strengthens cell–cell junctions. We show here for the first time that YAP activity plays a role in adhesion molecule-mediated mechanotransduction. It is a generally accepted concept that increased activation of FAK results in increased YAP/TAZ activity [17]. Our data indicate that this correlation might be more complex as pFAK levels in control and BRAF-depleted cells upon PECAM-1-mediated adhesion were similar, but YAP activation was different. It has been shown that actin disruption is a more potent modulator of YAP activity than the activity of myosin II [35]. Our data also indicate that MLC phosphorylation status inversely correlates with YAP activation upon force application (we found more active YAP in BRAF-depleted cells where the pMLC levels were decreased compared to control cells). An attractive mechanistic concept for YAP activation would be that angiomotin (AMOT), working as a YAP “inhibitor”, retains YAP in the cytosol [35]. In our experiments, an increased peripheral thickness also correlates with an increased nuclear localization of YAP. AMOT would be a possible candidate, which might mediate the localization of YAP in response to F-actin reorganization within the cell and this would determine the extent of YAP nuclear translocation during PECAM-1-mediated mechanotransduction. This hypothesis, however, needs further investigations.

## 4. Materials and Methods

### 4.1. Reagents

Primary antibodies for Western blotting (WB): BRAF, Cat# 14814S; ERK, Cat# 9102; pERK, Cat# 9101; GAPDH, Cat# 97166S; MLC, Cat# 8505S; pMLC (Thr18/Ser19), Cat# 3674S; VE-cadherin, Cat# 2500S; pYAP (Ser 127), Cat# 13008; all from Cell Signaling (Danvers, MA, USA).

Primary antibodies for immunofluorescence (IF): pMLC (Ser19), Cat# 3675S; PECAM-1, Cat# 3528S; VE-cadherin, Cat# 2500S; YAP (mouse), Cat# 12395; all from Cell Signaling; PECAM-1, Cat# MABF2033 from EMD-Millipore Corp. (Darmstadt, Germany); pFAK (Tyr397), Cat# 44-624G from Invitrogen (Waltham, MA, USA); pFAK (Tyr397), Cat# 611722 from BD Biosciences (Franklin Lakes, NJ, USA).

Secondary antibodies for WB: Peroxidase AffiniPure Goat Anti-Mouse IgG (H+L), Cat# 115-035-003; Peroxidase AffiniPure Goat Anti-Rabbit IgG (H+L), Cat# 111-035-003; all from Jackson ImmunoResearch (Ely, UK).

Secondary antibodies for IF: Chicken anti-Rabbit IgG (H+L) Cross-Adsorbed Secondary Antibody, Alexa Fluor 488, Cat# A21441; Goat anti-Mouse IgG (H+L) Cross-Adsorbed Secondary Antibody, Alexa Fluor 546, Cat# A11003; both from ThremoFisher Scientific (Waltham, MA, USA); Phalloidin CruzFluor™ 647 Conjugate, Cat# sc-363797 from Santa Cruz Biotechnology (Heidelberg, Germany); Phalloidin Alexa Fluor™ Plus 647 Conjugate, Cat# A30107 from Invitrogen.

siRNAs: siControl: ON-TARGETplus Non-targeting Control Pool, Cat# D001810-10-20; siBRAF: ON-TARGETplus Smart Pool Human BRAF, Cat# L-003460-00-0020; both from Dharmacon (Lafayette, CO, USA). siBRAF 3′-UTR set: siRNA sequences targeting the 3′-UTR region of BRAF were designed according to [36]. Two siRNA sequences were chosen and ordered from Merck Life Science (Sigma, Darmstadt, Germany): 5′-UACAUGAGCGAGACAUCCU-3′ and 5′-UAGAUCUGUUCAGUUUGCC-3′.

Chemicals for cell culture: basic Fibroblast Growth Factor, Cat# F0291; Gelatin, type B, 2% solution, Cat# G1393; Heparin, Cat# H3149; Hydrocortisone, Cat# H0396; Vitamin C, Cat# A4544; all from Sigma. Chemically Defined Lipid Concentrate, Cat# 11905031; DMEM, Cat# 10313021; EGF, Cat# PHG0311; GlutaMAX Supplement, Cat# 35050038; HBSS, Cat# 14025050; HEPES, 1M Buffer Solution, Cat# 15630049; Insulin-Transferrin-Selenium, Cat# 41400045; L-Glutamine, Cat# 25030081; MCDB-131 medium, Cat# 10372019; Penicillin/Streptomycin, Cat# 15140148; all from ThermoFisher Scientific (Waltham, MA, USA). Fetal Bovine Serum, Cat# P40-39500 from PAN Biotech (Aidenbach, Germany).

Inhibitors: BRAF inhibitor, PLX8394, Cat# S7965; FAK (pY397) inhibitor, Y15, Cat# S5321 and LATS inhibitor, TDI-011536, Cat# E1314 from Selleck Chemicals GmbH (Frankfurt am Main, Germany); MEK inhibitor, U0126, Cat# 662005 from Merck Life Science (Sigma).

HUVEC cell culture: HUVECs were purchased from Caltag Medsystems (Buckingham, UK) (Cat# ZHC-2301) and were cultured in MCDB medium, supplemented with 5% fetal bovine serum, 1% Penicillin/Streptomycin, 1% Chemically Defined Lipid Concentrate, 1% HEPES, 1% GlutaMAX Supplement, 0.3% Insulin-Transferrin-Selenium, 1 ng/mL basic Fibroblast Growth Factor, 2 ng/mL EGF, 5 µg/mL Vitamin C, 250 nM hydrocortisone and 7.5 U/mL heparin. Tissue culture dishes were coated with 0.5% gelatin for proper attachment of HUVECs. All cell culture was performed at 37 °C in a humidified atmosphere containing 5% CO_2_. For monitoring YAP localization, cells were kept in 50–50% MCDB-AIMV medium overnight; then, the next day the medium was exchanged for complete AIMV (containing 1% FBS, 1 ng/mL bFGF, 2 ng/mL EGF and 7.5 U/mL heparin). The medium was exchanged for another hour to a “reduced serum AIMV”, containing 0.1% FBS, 1 ng/mL bFGF, 2 ng/mL EGF and 7.5 U/mL heparin.

For immunofluorescence stainings, 8.5 × 10^4^ and 1.3 × 10^5^ cells were seeded on Imaging Dish CG 1.5 (Miltenyi Biotec, Bergisch Gladbach, Germany, Cat# 130-098-284) and on PELCO Clear Wall Glass Bottom Dishes (Ted Pella Inc. (Redding, CA, USA)), Cat# 14022-20), respectively. For Western blot analysis 4 × 10^5^ were seeded or Tissue Culture Dish (TPP, Trasadingen, Switzerland, Cat# 93040).

Inhibitor treatment: PLX8394, TDI-011536, U0126, and Y15 inhibitors were applied at a concentration of 1 µM, 5 µM, 10 µM and 15 µM, respectively, in complete MCDB. Cells were incubated with inhibitors at 37 °C for an hour prior to magnetic experiments.

### 4.2. Plasmid Cloning and Viruses

The plasmid of EGFP-BRAF was amplified from pcDNA-EGFP-BRAF [23] and was subcloned into the lentiviral destination vector (Addgene (Watertown, MA, USA)), Cat# 17454) using the NEB Builder kit (New England Biolabs (Ipswich, MA, USA)), Cat# E5520S). The cloning was verified by sequencing. Lentiviral supernatants were generated by co-transfection of HEK293T cells with a three-vector lentiviral system: using the specific expression vector combined with the lentiviral packaging and envelope plasmids pRSV-Rev, pMDLg/pRRE, and pCMV-VSV-G (kind gift of Prof. Guillaume Charras). Lentiviral packaging was performed as described previously [37] with a slight modification: the virus was concentrated by using the Lenti-X™ Concentrator (Takara Bio (San Jose, CA, USA), Cat# 631231) before mixing with the trypsinized HUVECs.

### 4.3. siRNA Transfection

siBRAF 3′-UTR set: siRNA sequences targeting the 3′-UTR region of BRAF were designed according to [36]. siRNA transfection was carried out in OPTI-MEM (ThermoFisher Scientific, Cat# 31985062) by using Lipofectamine RNAiMAX (ThermoFisher Scientific, Cat# 13778030) and siRNA (25 nM). Cells were incubated with the siRNA-RNAiMAX mixture for 4 h and seeded according to the type of the experiment.

### 4.4. PECAM-1 Antibody Binding to Magnetic Beads and Mechanotransduction

The antibody binding to magnetic beads was prepared according to Marjoram et al. [25]. Briefly, 20 µL 2.8 μm diameter tosyl-activated magnetic beads from Invitrogen (Dynabeads M-280 Tosyl-activated; Cat. #14203) were washed 3 times with 1 mL 0.1 M sodium phosphate buffer, pH 7.4. Finally, the beads were suspended in 100 µL buffer, transferred into a PCR tube, and 2.5 μg PECAM-1 antibody (Cat# MABF2033 from EMD-Millipore Corp.) was added. To produce a covalent linkage of protein to the beads the solution was incubated for 48–72 h at room temperature on a rotating wheel. Then, the beads were washed 3 times with 1 mL PBS and finally were suspended in 500 µL PBS.

Covalently cross-linked magnetic beads were diluted in complete MCDB (or in “reduced serum AIMV” for studying YAP localization) to obtain two to three beads for every HUVEC cell. Control plates were incubated with MCDB medium without beads, for studying the adhesion and mechanotransduction MCDB was supplemented with beads as described above. The beads were allowed to adhere to the cells for 20–25 min at room temperature. Normal dish lids were exchanged for lids containing Neodymium magnet, 2.5 cm × 0.5 cm disc or 3 cm × 0.5 cm disc, grade N52 (MagnetPlanet Webshop) and incubated for the indicated time points. The reaction was stopped by placing the dishes on ice and removing the lids with magnets. The culture medium was aspirated, and the cells were either fixed for immunofluorescence or washed with PBS and lysed for immunoblot analysis.

### 4.5. Immunofluorescence Staining of Fixed Monolayers

Cells grown on Imaging Dish were fixed with Image-iT™ Fixative Solution (ThermoFisher Scientific, Cat# R37814) for 15 min. After that, cells were washed with HBSS, permeabilized (0.25% Triton X-100 in TBS buffer containing 1% Tween (TBS-T), 10 min, room temperature), blocked (1% BSA in TBS-T, 1 h RT), and incubated with the primary antibodies (dilutions prepared in 1% BSA-TBS-T for VE-cadherin—1:400, pMLC—1:200, PECAM-1—1:2000, YAP—1:150 (Cell Signaling) or 1:500 (EMD Millipore Corp.), pFAK—1:150; incubation was performed overnight at 4 °C). After thorough washing in TBS-T, cells were stained simultaneously with the appropriate secondary antibodies (dilutions were prepared as 1:2000) and phalloidin (1:1000 in 1% BSA-TBS-T) for 1 h at RT, washed in TBS-T and PBS and also stained with Hoechst (Cat# 62249, ThermoFisher Scientific (Waltham, MA, USA)) for 5 min and finally washed in PBS prior to imaging. The measurements were carried out in an antifade solution, 1% DABCO 33-LV (Sigma, Cat# 290734), containing 50% glycerol in PBS. Confocal imaging was performed with a Nikon Ti2 inverted microscope (Expertline, Abberior Instruments, Göttingen, Germany). The field of view for imaging was a 120 µm × 120 µm area and pictures were taken by using a 60× lens (numerical aperture: 1.40, oil) at a resolution of 1000 × 1000 pixels.

### 4.6. Immunoblotting

Cells were harvested in 25 mM HEPES, pH 7.4, 150 mM NaCl, 1 mM EGTA, 1% NP-40, 10% glycerol, supplemented with the following protease and phosphatase inhibitors: 10 mM sodium pyrophosphate, 10 mM sodium fluoride, 5 mM sodium vanadate, 1 mM PMSF and cOmplete, EDTA-free protease inhibitor cocktail (Sigma, Cat# 4693132001). Lysates were centrifuged at 5000× *g* for 5 min at 4 °C and the supernatant was snap-frozen for further immunoblotting.

Proteins were separated using standard SDS-PAGE gel electrophoresis with 7.5% or 12% SDS-PAGE gels, transferred to PVDF membranes for immunoblot analysis using a wet blot transfer system (BioRad, Hercules, CA, USA), and stained with specific primary antibodies as indicated in each figure. After HRP-conjugated secondary antibody incubation, membranes were incubated with chemiluminescence substrate and developed on Hyperfilms. Bands were quantified using ImageJ 1.53c software (https://imagej.net/ij/download.html, accessed on 26 June 2020).

### 4.7. Immunoprecipitation

DMSO- or PLX8394-treated HUVECs (cultured to confluency on 10 cm TPP dishes) were harvested in 550 μL lysis buffer (for the content of the lysis buffer see Section 4.6). Lysates were centrifuged at 5000× *g* for 5 min at 4 °C and the supernatant was used for further immunoprecipitation. Then, 5 μL BRAF antibody (rabbit, Cell Signaling, Cat# 14814S) was added to 500 μL lysates and incubated overnight on a rotating wheel at 4 °C. Next morning, the lysate was briefly spun down and added to 40 μL 50% Rec-Protein G-Sepharose 4B Conjugate (ThermoFisher Scientific, #10-1241). The mixture was incubated for two hours on a rotating wheel at 4 °C. After that, the mixture was spun at 3200 rpm, washed three times with 1 mL lysis buffer (for each washing step), and eluted in sample buffer for further immunoblot analysis. RAF-1 (mouse, 610152, BD Biosciences) and BRAF (clone F7, mouse, Cat# sc-5284, Santa Cruz Biotechnology Inc.) antibodies were used for immunoblotting.

### 4.8. Intercelular Gap Formation Assay

Intercellular gap formation assay was carried out using a modified version of the permeability assay used in [38]. HUVECs were seeded on Glass Bottom Dishes pre-coated with 250 μg/mL biotinylated gelatin and were cultured in MCDB medium for 2 days. After cell treatment, the cells were fixed with Image-iT™ Fixative Solution for 15 min. After that, cells were washed two times with PBS and stained with Streptavidin-Alexa488 (2 μg/mL, Thermo Scientific) for 2 min. The cells were washed two times with PBS and labeled with Hoechst for 10 min, washed again two times with PBS, and kept in antifade solution until imaging was performed. Ten to fifteen pictures of each well were taken using a Nikon Ti2 inverted microscope (Expertline, Abberior Instruments, Göttingen, Germany). The field of view for imaging was a 425 µm × 425 µm area and pictures were taken by using a 20× lens (numerical aperture: 0.75, air) at a resolution of 1000 × 1000 pixels. Size of the stained area was determined on each image by quantitative image analysis using Fiji.

### 4.9. Transendothelial Migration Assay

Briefly, 6 × 10^4^ HUVECs were cultured on gelatin-coated 96-well inserts (8 μm pore size, Cat# 89089-938, VWR) for 48 h, starved overnight with 50–50% MCDB-AIMV medium, and then the medium was replaced with AIMV for 1 h. HUVECs were preincubated for 1 h with 1 μM PLX8394 or 10 μM U0126 or DMSO before applying melanoma cells on top of them. Before applying melanoma cells, the medium in the lower chamber was replaced by the growth medium of HUVECs.

A375 melanoma cells (1 × 10^5^) stained with Oregon Green dye (Cat# C34555, ThermoFisher Scientific) for 45 min were added to the upper chamber and after 15 min sedimentation, the cells were incubated for another 3.75 h with or without thrombin (3 U/mL). Transmigrated cells were dissociated from the lower part of the chamber by using a cell dissociation buffer (5 mM EDTA in PBS) and fluorescence intensities were measured on a CLARIOstar microplate reader (BMG LABTECH, excitation: 483 nm, emission: 530 nm). The integrity of the monolayers was determined by orange CellMask staining (Cat# C10045). Experiments were performed in triplicate.

### 4.10. Quantification of Colocalization

Quantification of the colocalization of pMLC and actin or actin and VE-cadherin or pMLC and VE-cadherin was carried out by using the Colocalization Colormap plugin in ImageJ [39]. This plugin calculates an index of colocalization where 1 stands for complete colocalization and 0 means lack of colocalization. Square area was determined at reorganized cell contacts and the index was calculated for each protein pairs.

### 4.11. Determination of the Number of Focal Adhesions

The number of focal adhesions was determined in 120 × 120 μm area. The threshold was set between 1.5 and 2.0% (in the case of pFAK antibody, rabbit host) or 3.5 and 4.0 (in the case of pFAK antibody, mouse host) and the image was converted to a binary one. As a result, the focal adhesions appear as white spots and the particles between sizes of 0.3 μm^2^ and infinity were counted using the Particle Analysis plugin in Fiji 1.53c software. To determine the number of focal adhesions at the cell periphery and inside the cell, the cell borders were assigned by PECAM-1 or VE-cadherin staining (Supplementary Figure S3). The periphery of the cells was defined 5 μm from the cell membrane by using the selection enlarge function of Fiji. For each cell, the number of pFAK spots were determined both at the cell periphery and in the cell center separately and then summed up for all cells having the same treatment in each experiment. Then, a ratio was calculated for both the cell periphery and the cell center related to the total number of focal adhesions (the sum of the pFAK spots determined for all cells, including the cell periphery and the cell center). After that, these ratios were used to calculate and show the distribution of the number of focal adhesions in a 120 × 120 μm area. We plotted the results of the three experiments (Figure 3d,i).

### 4.12. Quantification of the Relative Number of Remodeling (Discontinuous) Junctions

Discontinuous junctions were identified based on VE-cadherin staining. The number of discontinuous junctions (determined for cell pairs) were summed up and divided by the number of cells in each confocal image for the whole field of view. Then these numbers were plotted and compared.

### 4.13. Quantification of YAP Localization

Fluorescence intensities of YAP in the cytosol and in the nucleus were quantified in Fiji. In the composite image of YAP and Hoechst staining, the cell nuclei were determined and the sum of the fluorescence intensities of the nuclei was divided by the total fluorescence intensity from which the sum of the fluorescence intensities of the nuclei was subtracted. Since the beads give a signal in the YAP channel, these signals were also subtracted from fluorescence intensities obtained in the specific compartment.

### 4.14. Analysis of the “Apparent” Thickness of Peripheral Actin

At the cell periphery the intensity of actin was determined by the aid of the line tool in Fiji. The immunofluorescence images of VE-cadherin and actin were merged, and a line plot was generated. The distance between intensity minimums gave the thickness of actin bundles. The blue arrows on Supplementary Figure S1a–h indicate the thickness of peripheral actin. We have to note that these numbers are not showing the real size of the actin bundles, since they were quantified from confocal images; therefore, we term them “apparent” thickness. To measure the “real” thickness, we would have to take pictures with a superresolution microscope, such as STED, and, after deconvolution and fitting a Gaussian distribution profile, determine the “real” size of the bundles.

### 4.15. Statistical Analyses

Statistical analyses were carried out in GraphPad Prism 4.01 (https://www.graphpad.com/, accessed on 1 February 2005). Significance was determined by one-way or two-way ANOVA followed by a Bonferroni post-test. Differences between groups were considered statistically significant if *p* < 0.05.

## Data Availability

The original contributions presented in the study are included in the article/Supplementary Materials; further inquiries can be directed to the corresponding author/s.

## Supplementary Materials

The following supporting information can be downloaded at: https://www.mdpi.com/article/10.3390/ijms252011234/s1.
