# Supplementary material for: BRAF Modulates the Interplay Between Cell–Cell and Cell–Extracellular Matrix Adhesions in PECAM-1-Mediated Mechanotransduction
Source: Int J Mol Sci. 2024 Oct 18;25(20):11234. doi: 10.3390/ijms252011234 (PMC11508702; doi:10.3390/ijms252011234)

Supplementary Materials

BRAF Modulates the Interplay Between Cell–Cell and Cell–Extracellular Matrix Adhesions in PECAM-1-Mediated Mechanotransduction

Éva Gráczer [1], Katalin Pászty [1], Laura Harsányi [1], Csilla Lehoczky [2], Antónia Fülöp [3] and Andrea Varga [1,*]

[1] Department of Biophysics and Radiation Biology, Semmelweis University, H-1094 Budapest, Hungary
[2] Faculty of Information Technology and Bionics, Pázmány Péter Catholic University, H-1083 Budapest, Hungary
[3] Faculty of Electrical Engineering and Informatics, Budapest University of Technology and Economics, H-1111 Budapest, Hungary
* Correspondence: matkovicsne.andrea@semmelweis.hu

Figure S1, supplementary to Figure 1 Force-induced stress fiber formation is reduced in BRAF-depleted cells

Figure S2, supplementary to Figure 2 Inhibition of BRAF dimerization or MEK activity cannot phenocopy the effect of BRAF depletion

Figure S3, supplementary to Figure 3 BRAF depletion increases the number and distribution of pFAK positive cell-ECM adhesion sites

Figure S4, supplementary to Figure 4 FAK inhibition results in the weakening of cell-cell junctions in BRAF-depleted cells

Figure S5 supplementary to Figure 5 YAP activation in control cells prevents the force-induced remodeling of VE-cadherin junctions

Figure S1, supplementary to Figure 1 Force-induced stress fiber formation is reduced in BRAF-depleted cells

The thickness of peripheral actin was analysed in siControl- (**a-d**) and siBRAF- (**e-h**) transfected HUVEC monolayer without anti-PECAM-1 conjugated beads (**a** and **e**), with beads (**b** and **f**) and after application of two-minute (**c** and **g**) and five-minute (**d** and **h**) force. The immunofluorescence images of VE-cadherin and actin were merged, and a line plot was generated. The blue arrows indicate the thickness of peripheral actin. Representative images of the same experiment as in Figure 1**b-f** using a different siRNA for BRAF targeting the 3'-UTR region are shown on panels (**i**) and (**j**) (siControl) and (**k**) and (**l**) (3'-UTR siBRAF) for the adhesion and five-minute force application. The efficiency of the two different siRNA sets used was confirmed by western blotting (**m**).

In order to rescue the phenotype, we used the 3'-UTR siRNA to silence endogenous BRAF and transduced the cells to re-express EGFP or EGFP-BRAF (**n-p**) as indicated on the figure. BRAF knockdown cells with or without EGFP-BRAF re-expression were exposed to adhesion or five-minute force. Immunofluorescence images of VE-cadherin (**n**), actin (**o**) and EGFP (**p**) are shown, arrows indicate remodeling intercellular junctions in an EGFP-BRAF expressing cell upon force application.

Panel (**q**) displays western blot analysis of control or BRAF siRNA-transfected HUVEC monolayers applying either beads only or the application of two- or five-minute force. NA stands for no adhesion, A for adhesion, F 2' and F 5' two- and five-minute force application, respectively. Quantified results are plotted in panel (**r**), the amounts of pMLC were normalized to the total MLC. Results are shown from three independent experiments and were carried out at least from two different LOTs of HUVECs. Mean ± SEM was plotted, * denotes $p<0.05$.

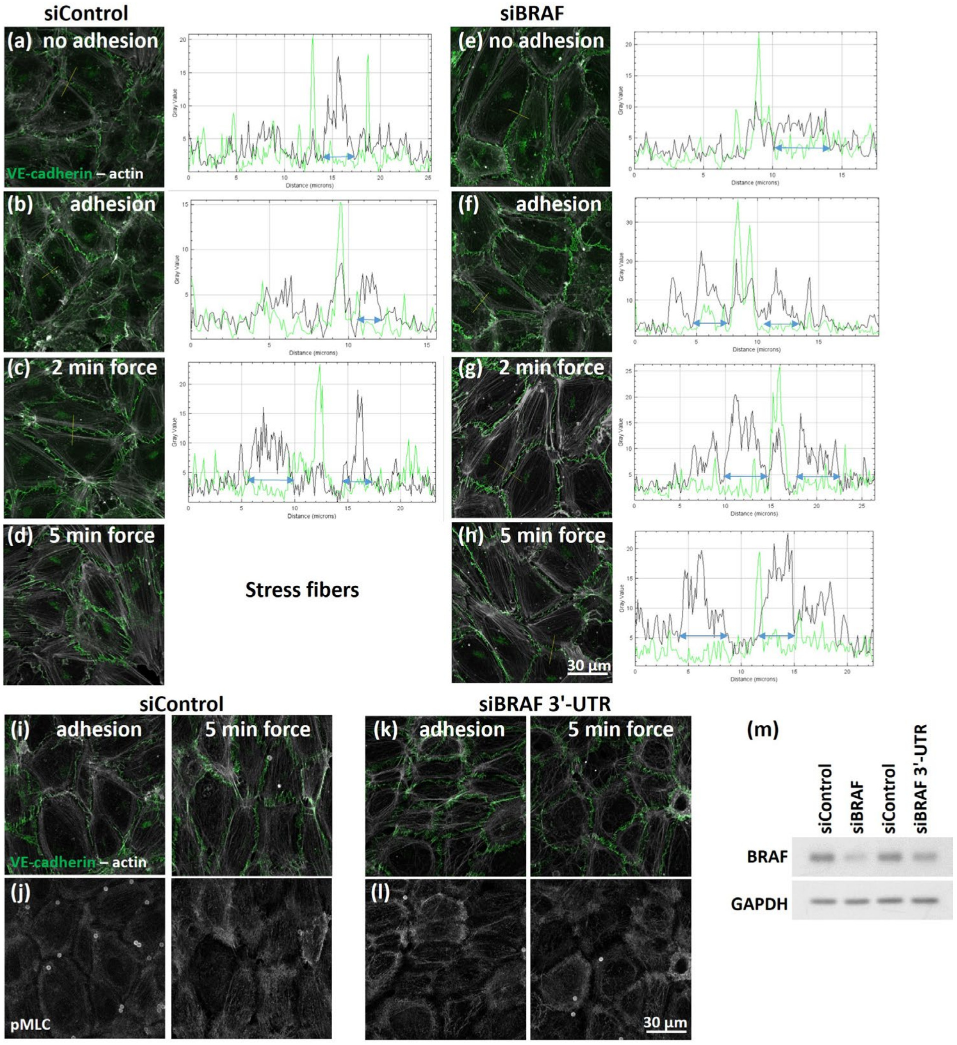

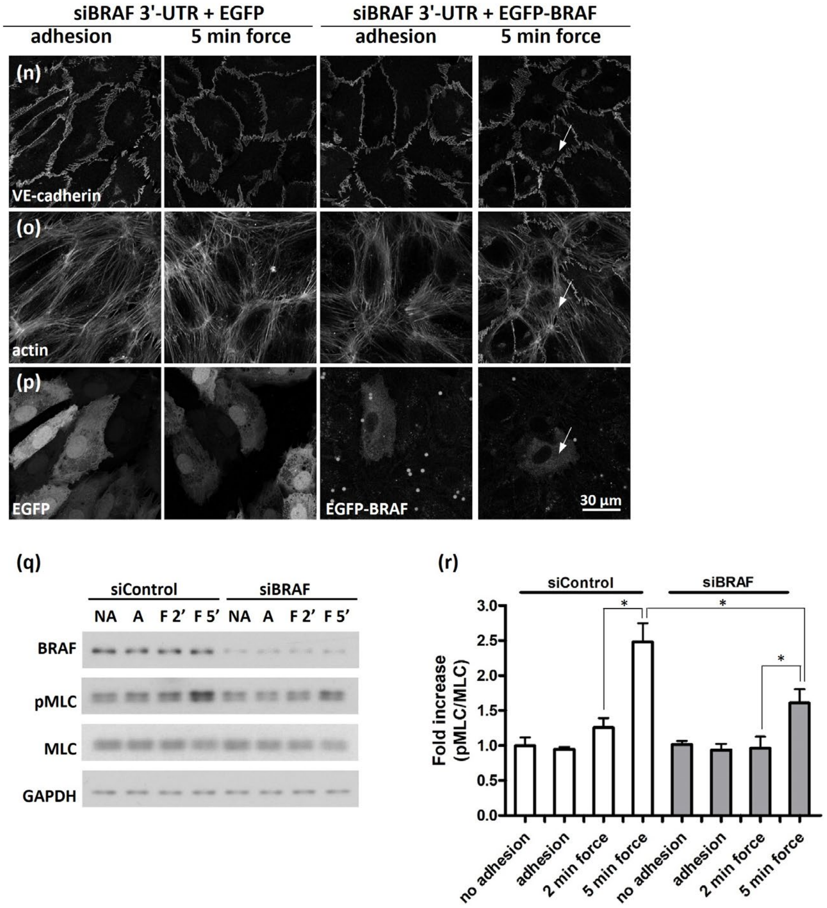

siBRAF 3'-UTR + EGFP
siBRAF 3'-UTR + EGFP-BRAF
adhesion
5 min force
adhesion
5 min force
(n)
VE-cadherin
(o)
actin
(p)
EGFP
EGFP-BRAF
30 µm
(q)
siControl
siBRAF
NA A F 2' F 5' NA A F 2' F 5'
BRAF
pMLC
MLC
GAPDH
(r)
siControl
siBRAF
Fold increase (pMLC/MLC)
3.0
2.5
2.0
1.5
1.0
0.5
0.0
*
*
*
no adhesion
adhesion
2 min force
5 min force
no adhesion
adhesion
2 min force
5 min force

Figure S2, supplementary to Figure 2 Inhibition of BRAF dimerization or MEK activity cannot phenocopy the effect of BRAF depletion

(**a**) Heterodimer formation between BRAF and RAF1 investigated by immunoprecipitation and quantified in control (DMSO) and PLX8394 treated cells. (**b**) Western blot analysis of the effect of U0126 treatment on MEK activity in DMSO and U0126-treated cells. Immunofluorescence images of control (DMSO-), BRAF-dimerization inhibitor, PLX8394- and MEK inhibitor, U0126-treated HUVEC monolayers are shown without adhesion of anti-PECAM-1 beads (**c**, **g**), with adhesion (**d**, **h**) or after application of force for two minutes (**e**, **i**) or five minutes (**f**, **j**). Cells were fixed and stained for VE-cadherin (green) and actin (grey; panels **c**-**f**), and pMLC (panels **g**-**j**). White arrows show cells with actin fibers decorated with pMLC. Representative images from two independent experiments are shown. Line scan analysis of the remodeling junctions (labeled by white arrows in Fig. 2**a**-**c**) are shown for VE-cadherin (green), actin (black) and pMLC (magenta) for the adhesion in control cells (**k**), PLX8394-treated (**m**), U0126-treated (**o**) cells or upon force application in control cells (**l**), PLX8394-treated (**n**), U0126-treated (**p**) cells. Western blot analysis of ERK and MLC phosphorylation in control, PLX8394- and U0126-treated samples are displayed on panel (**q**). Quantified results are plotted for pMLC (**r**), and for pERK(**s**). The amount of pERK and pMLC were normalized to the total ERK and MLC, respectively. Panel (**t**) shows the effect of BRAF-dimerization inhibitor, PLX8394 and the MEK-specific inhibitor, U0126 on the transmigration of A375 melanoma cells in the presence or absence of thrombin. Results shown are from two independent experiments and were carried out from two different LOTs of HUVECs. Mean ± SEM was plotted, * denotes $p<0.05$.

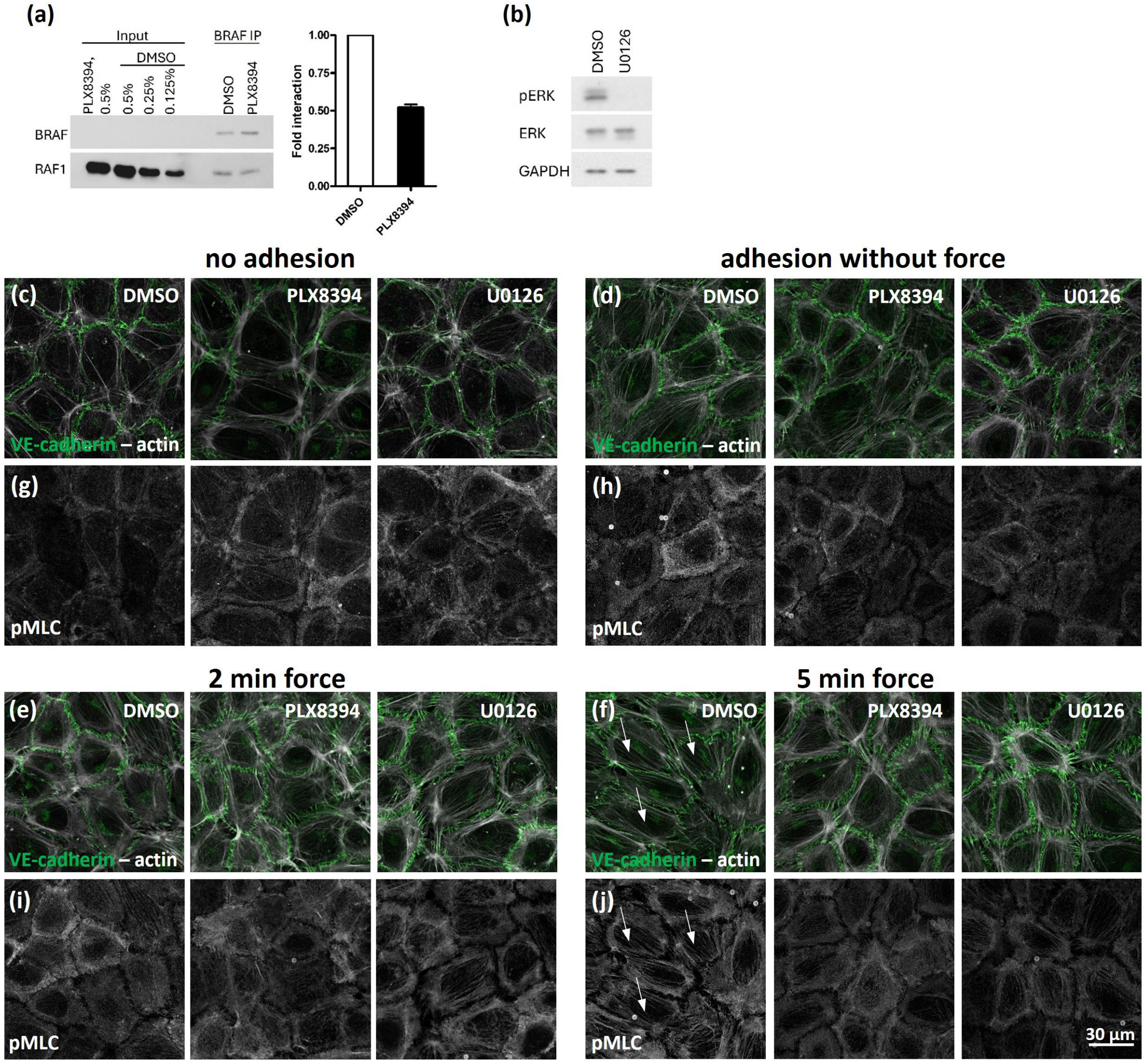

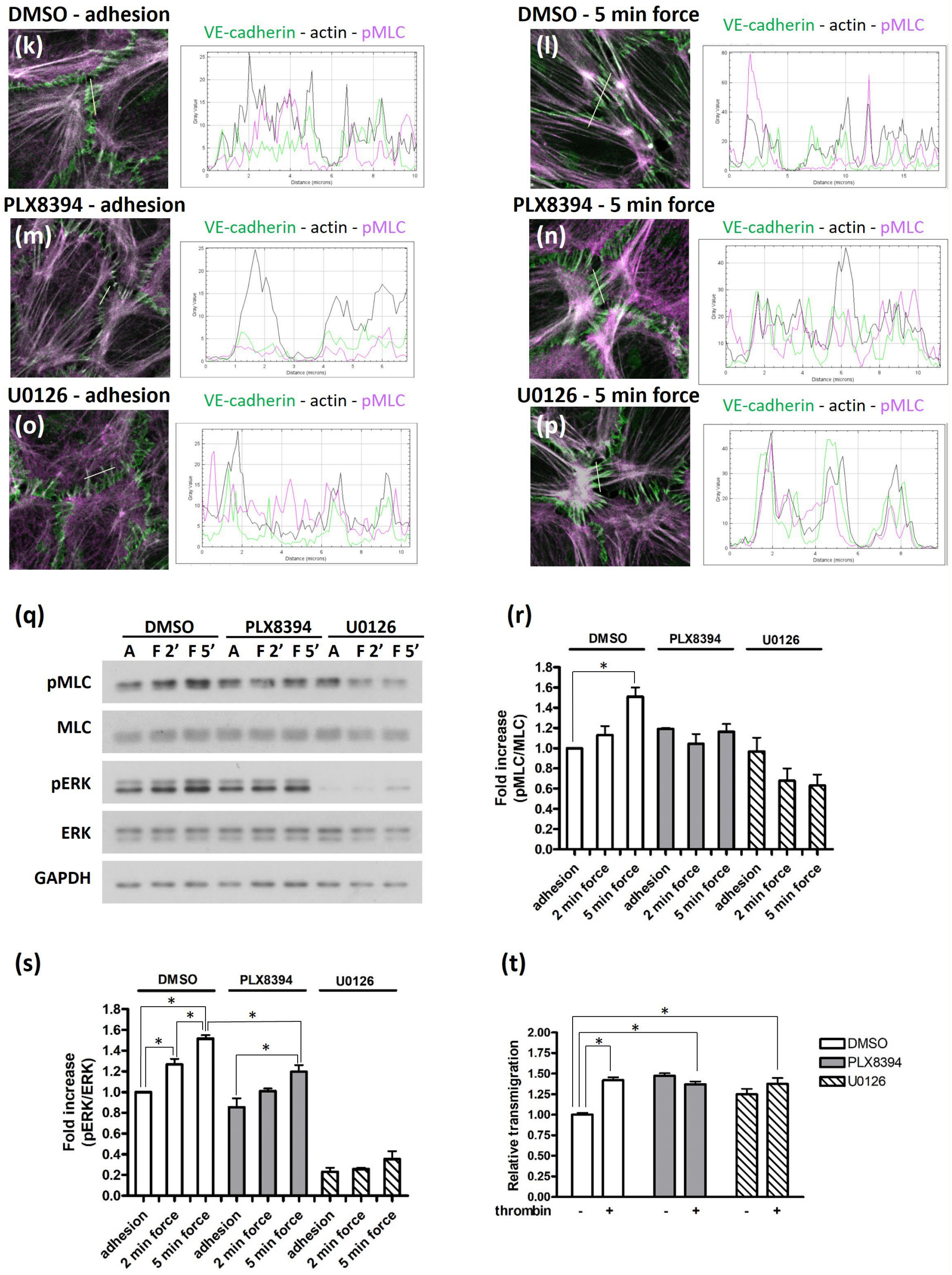

DMSO - adhesion
(k)
VE-cadherin - actin - pMLC
DMSO - 5 min force
(l)
VE-cadherin - actin - pMLC
PLX8394 - adhesion
(m)
VE-cadherin - actin - pMLC
PLX8394 - 5 min force
(n)
VE-cadherin - actin - pMLC
U0126 - adhesion
(o)
VE-cadherin - actin - pMLC
U0126 - 5 min force
(p)
VE-cadherin - actin - pMLC
(q)
DMSO
PLX8394
U0126
A F 2' F 5' A F 2' F 5' A F 2' F 5'
pMLC
MLC
pERK
ERK
GAPDH
(r)
DMSO
PLX8394
U0126
Fold increase (pMLC/MLC)
adhesion
2 min force
5 min force
(s)
DMSO
PLX8394
U0126
Fold increase (pERK/ERK)
adhesion
2 min force
5 min force
(t)
Relative transmigration
thrombin
DMSO
PLX8394
U0126

Figure S3, supplementary to Figure 3 BRAF depletion increases the number and distribution of pFAK positive cell-ECM adhesion sites

siControl- and siBRAF transfected HUVEC monolayers were exposed to adhesion by anti-PECAM-1 antibody-coated beads followed by five-minutes force application. Control and BRAF-depleted cells were fixed and stained for PECAM (**a**) and pFAK (rabbit host) (**b** and **c**), or a separate set of samples were fixed and stained for VE-cadherin (**d**) and pFAK (mouse host) (**e** and **f**). The border of the cells was designated based on either PECAM or VE-cadherin staining, then cells were divided to peripheral (5 µm from the border) and central part (**c** and **f**, labeled in yellow) to determine the number of pFAK spots in different parts of the cells.

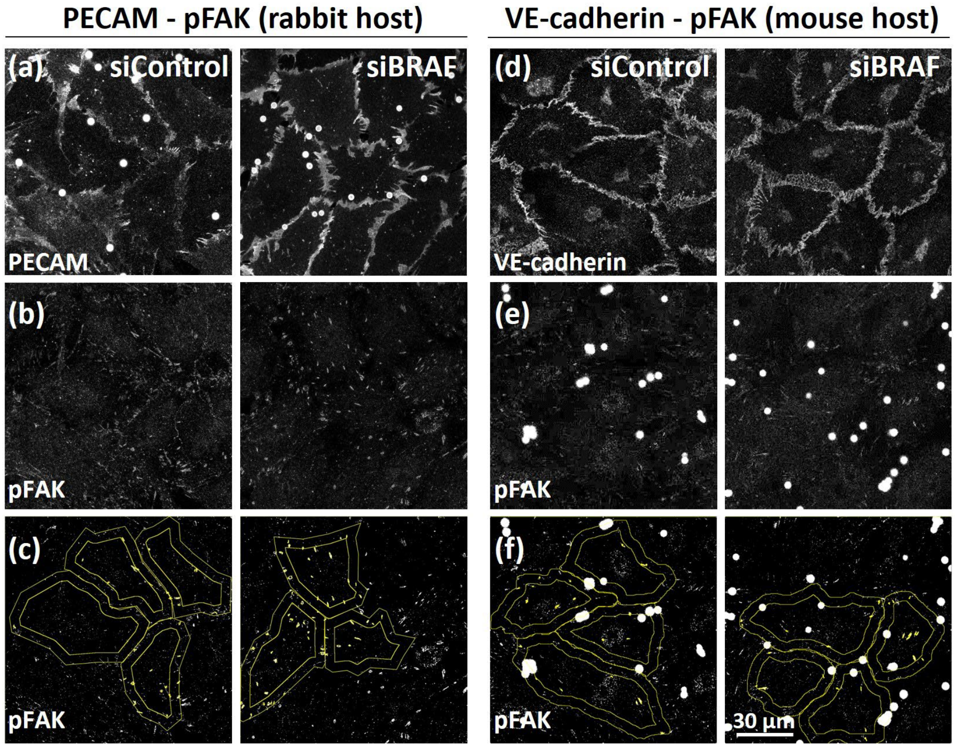

Figure S4, supplementary to Figure 4 FAK inhibition results in the weakening of cell-cell junctions in BRAF-depleted cells

Western blot analysis showing the effect of FAK inhibitor (Y15) on FAK phosphorylation in HUVEC cells treated with two types of BRAF siRNAs. A representative image of two independent experiments is shown. Quantified pFAK/FAK ratios are shown on the Figure, where siControl DMSO-treated cells were set to 1.00.

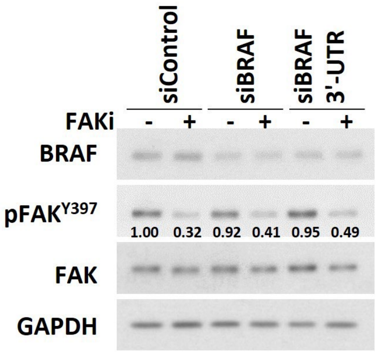

Figure S5 supplementary to Figure 5 YAP activation in control cells prevents the force-induced remodeling of VE-cadherin junctions

Western blot showing the effect of the specific LATS inhibitor TDI-011536 on the phosphorylation of the Ser127 residue of YAP.

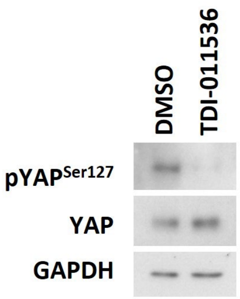

Supplement: Supplementary file 1 [file ijms-25-11234-s001.zip › ijms-3229440-supplementary.pdf]
